# Streamlined Schemes for Dosimetry of ^177^Lu-Labeled PSMA Targeting Radioligands in Therapy of Prostate Cancer

**DOI:** 10.3390/cancers13153884

**Published:** 2021-08-01

**Authors:** Jens Kurth, Martin Heuschkel, Alexander Tonn, Anna Schildt, Oliver W. Hakenberg, Bernd J. Krause, Sarah M. Schwarzenböck

**Affiliations:** 1 Department of Nuclear Medicine, Rostock University Medical Centre, 18057 Rostock, Germany; martin.heuschkel@med.uni-rostock.de (M.H.); alexander.tonn@uni-rostock.de (A.T.); anna.schildt@med.uni-rostock.de (A.S.); bernd.krause@med.uni-rostock.de (B.J.K.); sarah.schwarzenboeck@med.uni-rostock.de (S.M.S.); 2 Core Facility Multimodal Small Animal Imaging, Rostock University Medical Centre, 18057 Rostock, Germany; 3 Department of Urology, Rostock University Medical Centre, 18057 Rostock, Germany; oliver.hakenberg@med.uni-rostock.de

**Keywords:** PSMA, therapy, prostate cancer, dosimetry, single time-point

## Abstract

**Simple Summary:**

In patients with progressive metastasized castration-resistance prostate cancer PSMA radioligand therapies have shown promising results regarding clinical safety and efficacy. Dosimetry is mandatory due to legal regulations and also required for the estimation of doses to organs at risk allowing for individual tailoring of treatment in PSMA-RLT. Due to those factors and the often poor health status of patients which restricts intense dosimetric imaging protocols, there is a clear need for simplified dosimetric approaches in mCRPC patients treated with [^177^Lu]Lu-PSMA-617. In this study, we evaluated different dosimetric methodologies and found that a streamlined dosimetric approach is feasible and valid. This approach is based on single time-point imaging at 48 h p.i. in cycle 2 to 6 taking into account kinetic results of a full dosimetric scheme performed only in cycle1. These results might have a relevant impact on patients handling regarding dosimetry during [^177^Lu]Lu-PSMA-617 radioligand therapy.

**Abstract:**

(Background) Aim of this retrospective analysis was to investigate in mCRPC patients treated with [^177^Lu]Lu-PSMA-617 whether the absorbed dose (AD) in organs at risk (OAR, i.e., kidneys and parotid glands) can be calculated using simplified methodologies with sufficient accuracy. For this calculation, results and kinetics of the first therapy cycle were used. (Methods) 46 patients treated with 2 to 6 cycles of [^177^Lu]Lu-PSMA-617 were included. As reference (current clinical standard) full dosimetry of the OAR based on quantitative imaging (whole body scintigraphy and quantitative SPECT/CT at 2, 24, 48 and 72 h p.i.) for every cycle was used. Alternatively, two dosimetry schemes, simplified in terms of image acquisition and dose calculation, were established, both assuming nearly unchanged kinetics of the radiopharmaceutical for subsequent cycles. (Results) In general, for both OAR the simplified methods provided results that were consistent with the dosimetric reference method, both per cycle and in terms of cumulative AD. Best results were obtained when imaging was performed at 48 h p.i. in each of the subsequent cycles. However, both simplified methods tended to underestimate the cumulative AD. (Conclusion) Simplified dosimetry schemes are feasible to tailor multi-cycle [^177^Lu]Lu-PSMA-targeted therapies.

## 1. Introduction

Radioligand therapies (RLT) addressing the prostate-specific membrane antigen (PSMA), have shown most promising results regarding clinical safety and efficacy in patients with progressive metastasized castration-resistance prostate cancer (mCRPC) that are no longer responsive to treatments based on current guidelines [1,2,3,4,5]. Large prospective, randomized multicenter, phase II and III studies on the treatment of mCRPC patients with [^177^Lu]Lu-PSMA-617 (alone or in combination with other therapeutic agents) are under way (ENZA-p, NCT04419402; Thera-P, NCT03392428; VISION, NCT03511664). For nuclear medicine therapies dosimetry is required according to international regulations (e.g., 2013/59/Euratom [6]), recommendations of the International Commission on Radiological Protection (ICRP 140) [7] and national legislations. Dosimetry enables the estimation of doses to organs at risk (OAR) and tumor lesions which are known to show high intra- and inter-patient and intra-lesion variability due to different factors such as receptor density, binding affinity and tumor volume. In addition to legal regulations, individual assessment of doses to OAR is mandatory for a patient-specific approach in PSMA-RLT and might allow for the administration of higher cumulative activities, thereby increasing doses to the tumor and hence improving response to therapy significantly as recently shown by several studies [8,9,10,11,12]. Additionally, this position is supported by the EANM Guideline for PSMA-targeted therapies [13]. Dose estimation in clinical routine is generally performed according to the scheme defined by the Committee on Medical Internal Radiation Dose (MIRD) [14,15] using dedicated software tools [16,17]. Furthermore, voxel-wise approaches are increasingly used [9,18,19]. As these estimations have become part of the majority of clinical protocols of PSMA-targeted therapies [20,21,22], the use of a standardized imaging protocol is recommended [21]. Preferably, quantitative SPECT/CT imaging should be used, and in some cases, it can be accompanied by the more error-prone planar (whole-body (WB)) imaging [13]. Pharmacokinetics as well as patient compliance to tolerate multiple extensive imaging sessions must be considered when selecting appropriate imaging time-points and protocols. Particular attention must be given to the poor health status of most mCRPC patients, which limits imaging in clinical routine more than in patients treated with DOTATATE-therapies. Another factor, which is certainly not the main focus in clinical care, but which should not be neglected, is the incidence of prostate cancer which is approximately 40 times higher than for neuroendocrine neoplasia’s (NEN) [23,24]. Hence, the number of patients treated with PSMA-targeted therapies will be significantly larger [25] leading to increased utilization of imaging systems.

In light of this and what has already been proposed in several studies for Peptide Radio Receptor Therapies (PRRT) of NEN [26,27,28,29,30,31], optimization of intra-therapeutic imaging and dosimetry of PSMA-targeted therapies is important. Addressing this topic, Jackson et al. demonstrated in a recently published study that pharmacokinetic data extracted from a smaller cohort of patients treated with [^177^Lu]Lu-PSMA-617 could be applied to a much broader patient population to calculate dosimetry data from a single post-treatment scan. They calculated tissue-specific dose conversion factors for both, organs at risk and tumor lesions which can be used for this purpose [32]. Another interesting approach for single-time-point-based renal dosimetry, originally introduced by Hänscheid et al. for DOTATATE-therapies uses an approximate method to estimate the area under the time-activity curve [33]. The principle validity of this approach also for PSMA-targeted therapies was shown by Rinscheid et al. [34]. All of these studies showed that dosimetric calculations for the kidneys but also tumor lesions seem possible based on data from a single imaging time-point. However, comparisons on numerically larger cohorts (ideally in a multi-center setting) are necessary to show whether these promising results can be generalized.

In PSMA therapy, the kidneys are considered the main OAR, but the salivary glands should not be neglected either [35,36], whereas other organs such as the spleen and liver may not be considered as OAR. In contrast to previously published dosimetry data [37], lacrimal glands are not considered as OAR in clinical routine, also confirmed by recently published data [38].

Our retrospective analysis aimed to assess possible changes of the patient-specific kinetics (mainly characterized by effective half-life) in a multi-cycle setup. Additionally, we wanted to investigate whether based on the kinetics of the first cycle the total absorbed dose for all cycles in the OAR (kidneys and salivary glands), can be calculated with sufficient accuracy using simplified methodologies in mCRPC patients treated with multiple cycles of [^177^Lu]Lu-PSMA-617.

## 2. Materials and Methods

### 2.1. Patients

In our department [^177^Lu]Lu-PSMA-617 therapy is performed since autumn 2014. All patients gave written consent to undergo therapy with subsequent follow-up. Production and quality control of [^177^Lu]Lu-PSMA-617 (investigational product) was carried out according to GMP regulations. The detailed labeling procedure was described previously by Ahmadzadehfar et al. [39]. Therapies were indicated and conducted according to the German Medicines Law (AMG,§13[2b]), the current Declaration of Helsinki, paragraph 37 “Unproven Interventions in Clinical Practice” and the consensus recommendation of the German Society of Nuclear Medicine and corresponding inclusion and exclusion criteria [20]. Following the German radiation protection regulations, all therapies were implemented as in-patient treatment, and patients were hospitalized for at least 48 h. The continuation of the treatment was determined after each cycle of therapy based on the patient’s clinical presentation, biochemical parameters, response to treatment, the absorbed dose of OAR and consensus with uro-oncologist assessment and individual patient preferences. Each therapy cycle was accompanied by extensive imaging at 4 time-points depending on the compliance of the patient allowing for dosimetric calculations (see imaging and dosimetric schemes below).

From this data pool, data from patients with fully completed and evaluable post-therapeutic imaging after all cycles and at least 2 completed cycles of [^177^Lu]Lu-PSMA-617 therapy have been retrospectively included in this study. Based on these criteria, data sets of 46 patients (71.7 ± 7.9 years (mean ± standard deviation (SD)) treated with a mean applied activity of 6.01 ± 0.37 GBq of [^177^Lu]Lu-PSMA-617 per cycle were retrospectively enrolled in this study; for details on patients’ characteristics see Table 1. Out of these patients, 46/46 patients have received 2 cycles, 37/46 patients 3 cycles, 24/46 patients 4 cycles, 19/46 patients 5 cycles and 16/46 patients 6 cycles of therapy. Within the patient cohort, a very heterogeneous pattern of osseous, visceral and lymphatic metastasis was seen. Tumor burden varied from patient to patient.

The retrospective study design was presented to the ethics committee of the Rostock University Medical Center, and the need for a formal review was waived (file-no. A 2017-0152). The anonymized analyses were carried out in accordance with the declaration of Helsinki and its later amendments and the legal considerations of clinical guidelines.

### 2.2. Image Acquisition

For dosimetric calculations, a set of imaging consisting of whole-body scintigraphy (anterior and posterior planar imaging) and additional two-bed SPECT of the upper and lower abdomen at approximately 2, 24, 48 and 72 h after administration of [^177^Lu]Lu-PSMA-617 was acquired for all patients and all cycles. SPECT and whole-body imaging was performed on one of the systems of our department The exact details of the used SPECT systems and the applied acquisition and reconstruction protocols are shown in Table 2. On day 2, SPECT acquisition was followed by an auxiliary CT scan for CT-based corrections (attenuation correction, Monte-Carlo-based scatter correction) and organ delineation. If the gamma camera IRIX was used, the CT was acquired on the Symbia T6. SPECT images were quantitatively reconstructed using an OSEM reconstruction protocol (Hybrid Recon 3.0, Hermes Medical Solutions); for details see also Table 2.

The reconstruction workflow included a co-registration step using a rigid body algorithm, to align SPECT and CT data before the application of all CT-based corrections within the reconstruction algorithm. For quantitative SPECT imaging, all SPECT systems were calibrated by determining a camera-specific calibration factor using the methodology recommended by the MIRD pamphlet No. 26 [40]. This included also the CT calibration using an electron density phantom (model 062M, CIRS, Norfolk, VA) enabling precise conversion of CT data in Hounsfield units (HU) to the linear attenuation coefficient, μ (cm^−1^) [41]. Activity recovery coefficients as a function of the volume have been determined according to the mentioned MIRD pamphlet No. 26.

For planar whole-body (WB) imaging a patient specific-syringe, filled with approximately 20 MBq lutetium-177 at the first imaging time point, was placed as standard between the ankles of the patients allowing for normalization and quantification applying the methodology proposed by Bailey et al. [42]. Additionally, images were further processed using the conjugate-view method and scatter correction based on the triple energy window technique [43]. However, since the salivary glands are close to the body surface, the application of the attenuation correction of the planar images using an additional scan with a flat source phantom was omitted to minimize patient distress.

### 2.3. Dosimetric Data Analysis

Calculation of the organ-specific absorbed dose (*AD*) for kidneys and salivary glands was performed under the assumption of a uniform distribution of activity in the organs, and based on the scheme established by the Radiation Dose Assessment Resource (RADAR) Task Force of the Society of Nuclear Medicine using Olinda 2.1.1 [16,44]:

(1) AD=N×DF

where AD represents the absorbed dose of the specific organ, *N* is the number of nuclear disintegrations in the source organ and *DF* is a dose conversion factor which is radionuclide specific and geometry dependent. Co-registration and VOI drawing were performed using Hybrid Viewer (V 5.0.3; Hermes Medical Solutions). Dosimetric calculations for the kidneys and the salivary glands were based on quantitative SPECT and planar WB imaging, respectively. Volumes of interest (VOI) for the kidneys were manually drawn based on the CT, copied to the corresponding SPECT series and the time- and organ-specific activity was extracted and corrected by appropriate activity recovery coefficient. Patient-specific masses of the kidneys, used for mass scaling of the *DF*, were derived by multiplying individually determined CT volume of the kidneys by the generalized organ-specific densities published in Annex A of ICRP Publication 110 [45].

The parotid glands were used as a surrogate for salivary glands since these are considered to be particularly sensitive to radiation [36]. Delineation of the parotid glands was performed on a combined anterior-posterior calibrated WB image (result of the quantitative correction described above) using a slightly oversized region of interest (ROI) to include all relevant activity. ROI for background correction was placed in the vicinity. For each patient, the same mass (m = 14.3 g) was used for the parotid glands [46].

Time activity curves (TAC) for both organs were composed of two functions: a linear function from time 0 (start of the infusion) to the first time-point of imaging (representing a rapid uptake of the [^177^Lu]Lu-PSMA-617) and a bi-exponential fit to the 4 imaging time-points. Curve fitting was performed by in-house coded and validated LabVIEW apps (ver. 2017, Nat. Instruments), based on the Levenberg-Marquardt nonlinear least-squares algorithm. The goodness of fit was assessed by the coefficient of determination R^2^. The effective half-life of the elimination phase (*eHL*) was extracted and complete organ-specific functions were analytically integrated to infinity to calculate cumulated activities and the number of disintegrations (*N*) which were transferred to OLINDA for dose calculation applying the ICRP 89 Adult Male Reference Phantom [47]. For the estimation of the *AD* to parotid glands, the sphere model implemented in OLINDA was used [48]. The results were scaled by the injected activity to calculate the total *AD* (in units of Gy) of the specific organ.

Based on this general procedure to derive organ-specific AD from the series of 4 time-points of one cycle three different methods were explored to calculate patient- and organ-specific *AD* for multiple therapy cycles.

#### 2.3.1. Method 1 (M1)

For each cycle, *AD* was calculated according to the methodology described above based on the imaging data available for all 4 time points, hereinafter referred to as ‘full dosimetric scheme’.

#### 2.3.2. Method 2 (M2)

**Figure 1:**
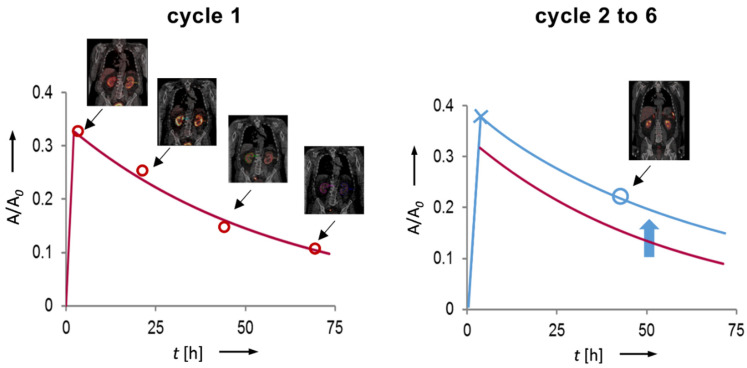
Principle of the scaling of the time-activity curve of cycle one based on a single time point measurement in the following cycles (in this example approx. 48 h p.i.). The TAC is corrected for cycle-specific differences in organ uptake, while maintaining the kinetic behavior derived in cycle one.

*N* and *AD* of the OAR for the first cycle were calculated according to method 1 and the organ-specific TAC were extracted. For the following cycles, these TAC were scaled based on the organ activities extracted from one single time point (planar whole body for salivary glands and a single SPECT/CT image for kidneys), assuming the same pharmacokinetic behavior, see Figure 1. Corrected TAC have been integrated till infinity and the resulting *N* were transferred to OLINDA and the *AD* of the specific organ was derived.

Quantification of the activities in the kidneys and salivary glands at the single imaging time was performed in the same manner as in cycle one. This scheme was studied under the assumption that imaging is only available for the time point 24 h, 48 h or 72 h p.i. The organ-, time- and cycle-specific *AD* thus calculated were designated according to the following scheme: $AD_{o\_Cx\_y}$, where *o* is kidney (Ki) or parotid glands (PG), *x* is the cycle from 2 to 6 and *y* is the single imaging time point (24, 48 or 72 h).

#### 2.3.3. Method 3 (M3)

The AD of the OAR for the first cycle was calculated according to method 1. The *AD* of the OAR for one of the following cycles (*x*) was estimated by simply multiplying the *AD* of the first cycle with the ratios of administered activities of cycle *x* and the first cycle [28] according to

(2) $$AD_{x}=AD_{1}\frac{A_{x}}{A_{1}}$$

### 2.4. Statistical Analysis

The results of dosimetry applying the full dosimetric scheme for each patient and each cycle according to method 1 were assumed to be the most precise one and were used as the reference for comparison and correlation with the results of the other two methods. To compare *eHL* and *AD* to kidney and parotid glands in M1, M2 and M3 for all cycles but also for every single cycle Bland-Altman analysis was used as well as repeated measures analysis of variance (rmANOVA). Results of Bland-Altman analysis were derived in terms of limits of agreement (LoA) calculated as the mean difference ± 1.96 x standard deviations. Furthermore, the total *AD* over all cycles, each calculated by the 3 different methods, were estimated and compared. Possible differences were evaluated for magnitude and significance. Statistical analysis was performed using Prism 9 (GraphPad Software Inc., San Diego, CA, USA). Significance was assumed in the case of *p* < 0.05. All values are given as mean ± SD unless otherwise indicated.

## 3. Results

Mean administered activity over all cycles was 6006 ± 365 MBq. No statistically significant differences were found regarding administered activities neither patient- nor cycle-specific (*p* = 0.148, *p* = 0.118, respectively).

### 3.1. Absorbed Doses Calculated Using Method 1

Applying the full dosimetric scheme, the mean absorbed kidney dose and dose coefficient slightly increased between the first therapy cycle and the sixth therapy cycle (2.98 ± 1.32 vs. 3.75 ± 0.79 Gy; 0.50 ± 0.22 Gy/GBq vs. 0.63 ± 0.13 Gy/GBq) without showing a statistically significant difference regarding variance (*p* = 0.139). Similar results were found for the *eHL* with 38.1 ± 14.5 h in cycle 1 and 41.7 ± 15.2 h in cycle 6 (*p* = 0.355). Similarly, for the parotid glands no statistically significant differences of *AD*, dose coefficient or *eHL* were found for the first and sixth cycle (4.77 ± 2.21 Gy vs. 4.82 ± 2.18 Gy, *p* = 0.284; 0.79 ± 0.37 Gy/GBq vs. 0.79 ± 0.35 Gy/GBq, *p* = 0.148; 33.16 ± 13.24 h vs. 33.89 ± 11.86 h; *p* = 0.262).

**Figure 2:**
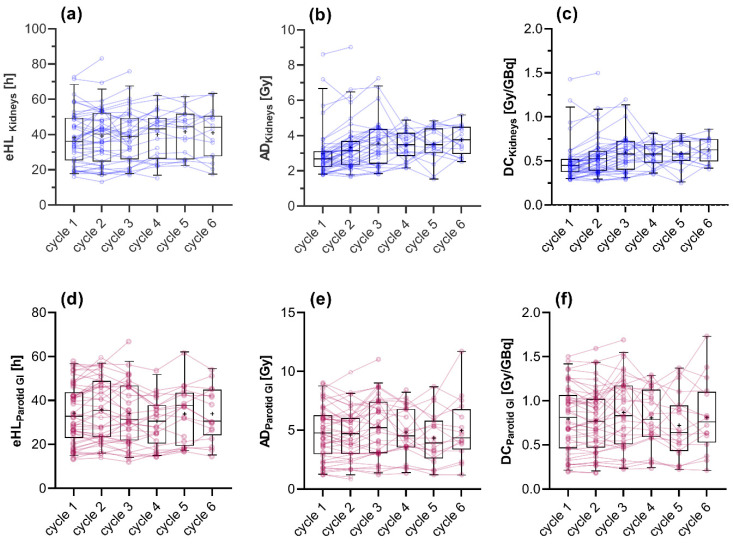
Boxplots showing the quartiles, the 5th and 95th percentiles (whiskers) and the mean (+) of the **e**ffective half-life (*eHL*), absorbed doses (*AD*) and dose coefficients (DC) for the kidneys (**a**–**c**) and the parotid glands (**d**–**f**). The colored dots represent the course of the values for the respective patients.

The highest *AD* per cycle in our cohort were 9.0 Gy (5.4 GBq administered activity) and 10.9 Gy (6.4 GBq administered activity) for the kidneys and parotid glands, respectively. A complete overview summarizing all of the above-mentioned parameters for kidneys and parotid glands for every cycle is given in Figure 2 and Table 3.

### 3.2. Absorbed Doses Calculated Using Method 2

**Figure 3:**
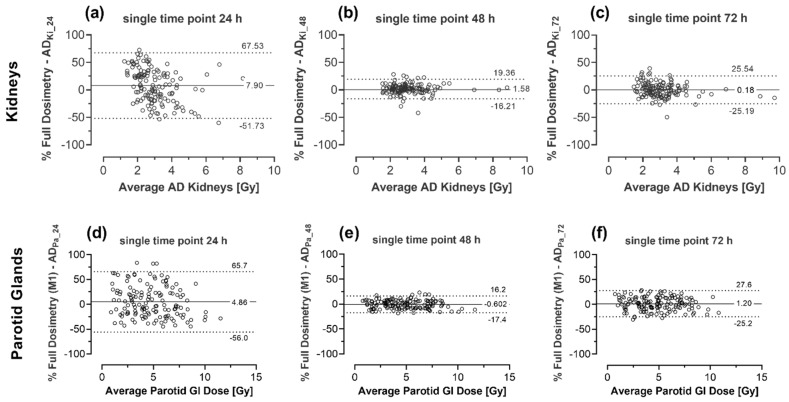
Bland-Altman-Analysis comparing calculated absorbed doses using full dosimetric scheme 1 (used as reference) and streamlined dosimetry (method 2) for single time-point imaging at 24, 48 and 72 h p.i. for kidneys (**a**–**c**) and parotid glands (**d**–**f**). The solid line shows the mean bias between the two methods, whereas dotted lines show the 95% limits of agreement.

**Figure A1:**
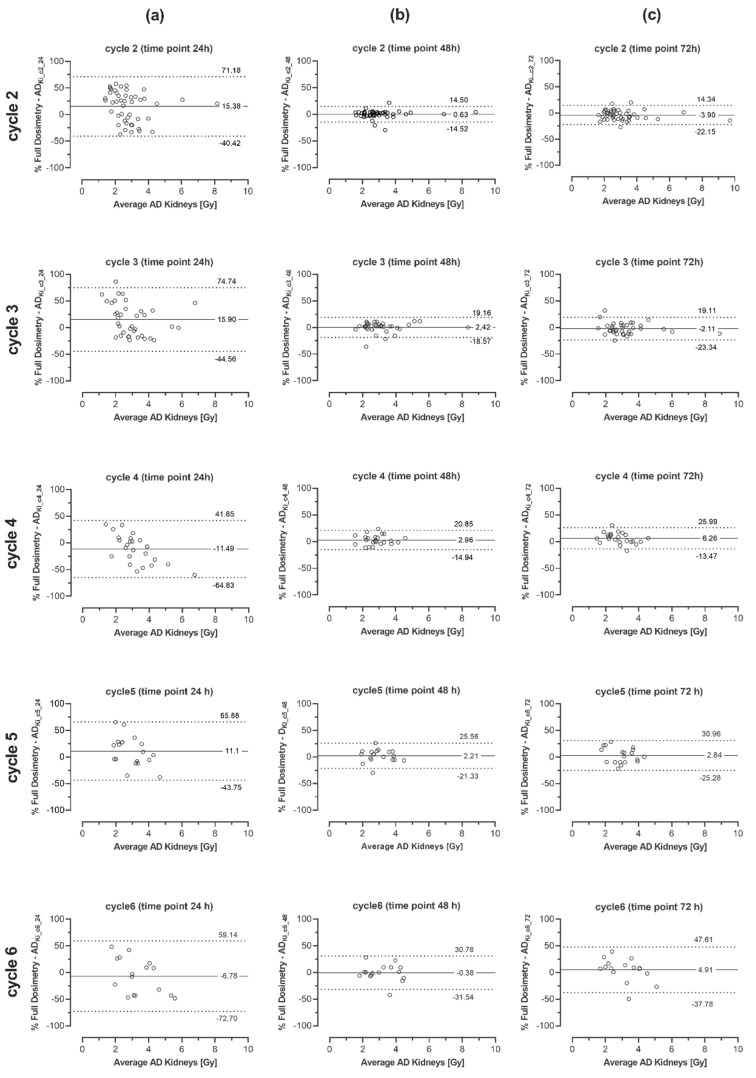
Bland-Altman Analysis comparing calculated absorbed doses of the kidneys using full dosimetric scheme (method 1, used as reference) and streamlined dosimetry (method 2) for therapy cycles 2 to 6 and each single time-point imaging at 24 (**a**), 48 (**b**) and 72 h p.i. (**c**).

**Figure A2:**
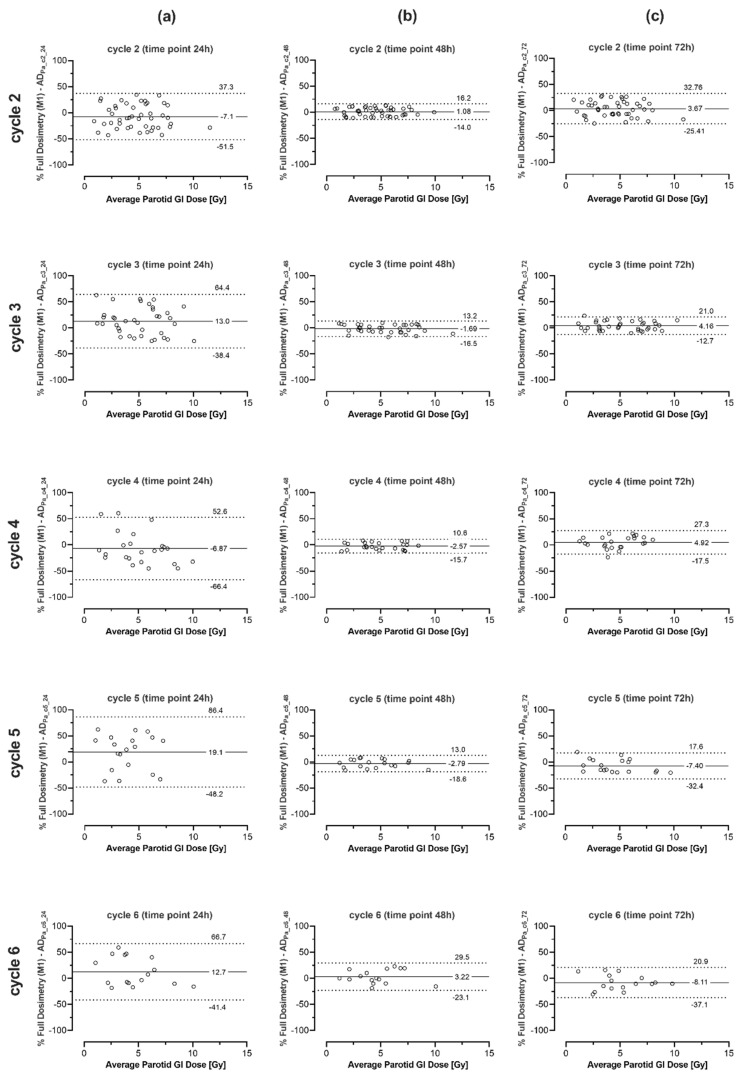
Bland-Altman Analysis comparing calculated absorbed doses of parotid glands using full dosimetric scheme (method 1, used as reference) and streamlined dosimetry (method 2) for therapy cycles 2 to 6 and each single time-point imaging at 24 (**a**), 48 (**b**) and 72 h p.i. (**c**).

Compared to method 1, the Bland-Altman analysis of all cycles among the three time points used within the simplified dosimetric scheme (i.e., 24 h vs. 48 h vs. 72 h p.i.) showed that the smallest range of differences of absorbed kidney dose was achieved using single time-point imaging at 48 h p. i. The LoA was approximately −1.6% ± 17.8%. For single imaging at 24 h p.i. LoA was 7.9 ± 59.6% and at 72 h LoA was 0.18 ± 25.3%. Comparable results were found for parotid glands: LoA were 4.9 ± 61.0%, 0.6 ± 15.8% and 1.2 ± 26.3% for single time-point imaging at 24, 48 and 72 h, respectively. Figure 3 depicts this in more detail. Similar results for both OAR were found in the Bland-Altman analysis of each therapy cycle (cycle 2 to 6). Results for this detailed analysis are shown in Table 4 and depicted in the Appendix A (Figure A1: kidneys and Figure A2: parotid glands).

### 3.3. Absorbed Doses Calculated Using Method 3

**Figure 4:**
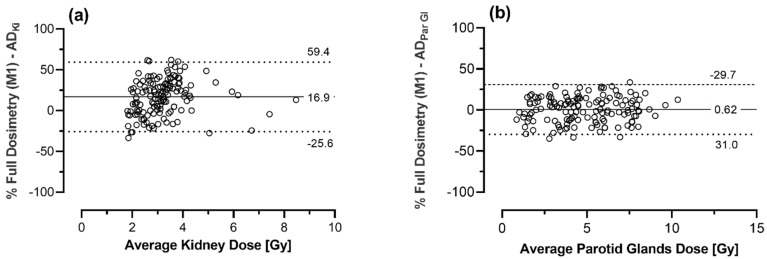
Bland-Altman-analysis comparing calculated absorbed doses using full dosimetric scheme (method 1, used as reference) and dosimetry based on calculated absorbed doses of cycle 1 and adaptation for applied activities (method 3) for kidneys (**a**) and parotid glands (**b**). The solid line shows the mean bias between the two methods whereas dotted lines show the 95% limits of agreement.

The estimated *AD* for kidneys and parotid glands using M3 and the mean differences compared to the results using method 1 are summarized in Table 5. Bland-Altman analysis revealed a LoA of approximately −16.9 ± 42.5% and −0.6 ± 30.4% for the kidneys and parotid glands, respectively, see also Figure 4.

### 3.4. Comparison of Total Absorbed Doses

The total *AD* for kidneys and parotids were as follows: When using M1 for dosimetry, the total *AD* for the kidneys over all cycles and patients was 13.7 ± 6.8 Gy. For those patients who completed 6 cycles, the absorbed renal dose was 20.7 ± 3.3 Gy. For parotid glands, the total *AD* over all cycles and all patients was 19.6 ± 11.53 Gy and 28.3 ± 11.9 Gy for those patients who completed 6 cycles. The highest total *AD* in our patient cohort was found in patients who completed 6 therapy cycles with 26.4 Gy and 47.1 Gy for kidneys and parotid glands, respectively.

**Figure 5:**
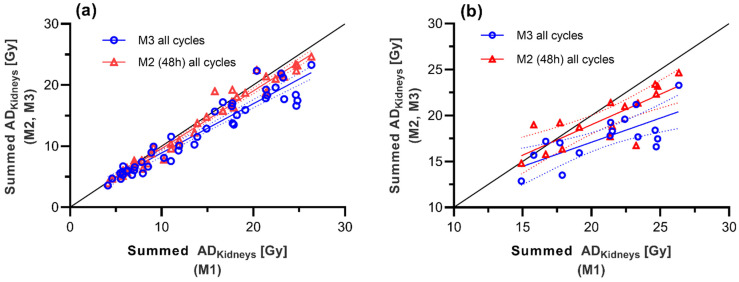
Correlation of the estimated absorbed dose of the kidneys calculated by using M1 (*x*-axis) vs. M2 and M3 (*y*-axis) for all cycles (**a**) as well for the sub-group of patients having received all 6 cycles (**b**). The dotted line represents the line of identity.

When calculating the total dose to the OAR over all cycles using M2 and M3, *AD* was underestimated for the kidneys as well as the parotid glands. Figure 5 shows this using the kidneys as an example for both methods and compares it to M1. Comparing M2 to M1 for all 6 cycles the mean difference for total *AD* of the kidneys was −3.9 ± 6.1%, with an underestimation of up to −21.1% and an overestimation of up to 11.3%; differences were not statistically significant (*p* = 0.102). Comparing M3 and M1 an even higher underestimation was seen: −15.2 ± 11.5% (min: −32.8%, max: 15.2%); differences were statistically significant (*p* < 0.001 **). The results for parotid glands were similar. The mean difference comparing M2 to M1 for total *AD* of the parotid glands was approximately −6.2 ± 9.1% with an underestimation of up to −19.8% and an overestimation of up 17.6%; differences were statistically significant (*p* < 0.001 **). As for the kidneys, a comparison of M3 and M1 showed an even higher and significant underestimation of total *AD* of the parotids: −15.3 ± 10.3% (min: −37.1%, max: 21.3%). These differences were also statistically significant (*p* < 0.001 **).

## 4. Discussion

Personalized dosimetry in [^177^Lu]Lu-PSMA RLT is crucial regarding the need for individualized and optimized treatment on the one hand and limitations of doses to OAR on the other hand as recently discussed by Stabin et al. [49]. The doses to OAR must not exceed the clinically established limits. However, those limits are derived from external beam therapy and are currently under debate because of their limited transferability to RLT. In contrast with external beam radiotherapy, RLT with Lutetium-177 has the biological advantage of delivering a low dose rate of beta radiation thereby maximizing the opportunity for normal tissue repair and minimizing late radiation damage. In a study by Bergsma et al. [50] on the renal radiation toxicity risk from [^177^Lu]Lu-DOTATATE no grade 3 or 4 renal toxicity with a 24 Gy cumulative kidney dose was observed. In the small group of patients having received doses greater than 28 Gy no grade 3/4 toxicity or an annual reduction of creatinine clearance greater than 10% occurred. Consequently, the authors suggested an elevation of the 28 Gy dose limit. Considering the comparable kidney dosimetry profile for [^177^Lu]Lu-PSMA-617 and [^177^Lu]Lu-DOTATATE due to the renal clearance of both drugs as well as their target expression on the renal proximal tubules and for DOTATATE also on the glomeruli, the increased dose limit could also be applied to PSMA-targeted therapy.

The salivary glands are also highly radiosensitive organs, therefore dose limits for external beam radiation-induced salivary gland damage have been well-defined [51,52]. The reduction in gland function gradually increases at absorbed doses of 20–40 Gy with a strong reduction (usually by >75%) at >40–46 Gy [51,53]. However, a recent review by Heynickx et al. [36] discussed the limited knowledge about the damage to the salivary glands by PSMA-targeted therapies and the causative mechanisms. One of the conclusions was that more studies including valid treatment-emergent dosimetry are necessary to obtain reliable dose-response relationships and to define dose limits for the salivary glands. In our study with its small patient sample doses are in line with previously published studies [3,21,54] with mean *AD* of kidney and salivary glands far below critical limits even after six therapy cycles of [^177^Lu]Lu-PSMA-617.

However, some patients showed a high *AD* to kidneys resulting in termination of treatment, mainly caused by already initially impaired kidney function, characterized by reduced eGFR. This finding is in accordance with the results of a study by Sandström et al. [55] on individualized dosimetry of kidney and bone marrow in patients undergoing [^177^Lu]Lu-DOTA-octreotate treatment. In this study, 20% of patients could only be treated with fewer than 4 therapy cycles due to limiting doses to OAR. These data confirmed the need for personalized dose calculation to avoid unacceptably high doses to OAR as well as undertreatment.

The current gold standard of dosimetry consists of a full dosimetric scheme for every cycle which is, however, clinically challenging due to the poor health status of most mCRPC patients. Therefore, simplified methods are needed. Willowson et al. [29] showed the feasibility and accuracy of single time-point imaging for renal dosimetry following [^177^Lu]Lu-DOTATATE therapy. According to the results of our study, the pure extrapolation based on *AD* derived from full dosimetric scheme only for the first cycle (method 3) showed an acceptable correlation with the results of method 1. However, absorbed doses of the kidneys and the parotid glands regarding all cycles were on average underestimated by −15% (kidneys and parotid glands) and in some cases even by up to −32% and −37 for kidneys and parotid glands, respectively. Underestimation increased in later cycles suggesting limited clinical usability. In contrast, method 2 with single-time-point imaging at 48 h p.i. in cycle 2 to 6 can be considered as a reliable alternative whose results showed a significant correlation with the *AD* of the full dosimetric scheme (method 1). Additionally, the difference in *AD* between method 1 and 2 were in an acceptable range of approximately ±6% and ±10% for kidney and parotid glands, respectively. In addition, single time-point imaging after 72 h has shown acceptable differences in *AD* to the reference method M1. The larger differences in *AD* might be caused by the outliers (mainly in cycles 5 and 6) which could be a consequence of the smaller sample sizes (see Appendix A; Figure A1 and Figure A2). The promising results of single time-point imaging at 48 h p.i. (being in the range of the effective half-life) are in line with the study by Hänscheid et al. [33]. In this study, the authors showed that the time-point of imaging is most appropriate if close to the effective half-life which is also the case in our study.

The increasing differences of *AD* between method 1 and 2, the non-significant increase of *AD* over the cycles, and the increasing effective half-lifes in kidneys (as previously also shown by Garske et al. [26] in [^177^Lu]Lu-octreotate therapy) between cycle 2 and 6 suggest the need to repeat full dosimetry (at least once) during multiple therapy cycles. However, given the small patient sample in our study, we could not address this aspect in our study and additional data are needed to investigate this issue. The observed non-significant but measurable increase in renal *AD* with increasing number of therapy cycles might be caused by the tumor sink effect as discussed in the literature [56,57,58,59], which is characterized by an increased absorbed dose to the kidneys due to a decreased tumor volume. However, since we did not systematically assess the total tumor volume (TTV) and its therapy-related changes in our study, we have to stick to a purely descriptive characterization at this point. This issue should be addressed in further studies.

Limited data are available regarding simplified dosimetry of [^177^Lu]Lu-PSMA RLT. Rinscheid et al. [34] demonstrated that a single SPECT/CT measurement at 52 h p.i. yielded good approximations for the time-integrated activity coefficients of the kidney, consistent with the results of our study. Jackson et al. [32] presented conversion factors to estimate the absorbed dose from a measured activity concentration in the treatment of prostate cancer with [^177^Lu]Lu-PSMA-617. They showed that the minimum uncertainty using the single-time-point model for parotid glands occurs at approximately 48 h after administration. The authors concluded that the ideal imaging time to yield accurate dose estimates across tissue types is in the window of 2 to 3 d after administration of [^177^Lu]Lu-PSMA-617 which is—despite different methodology—in line with the results of the presented study.

In our study, we did not calculate the dose to red bone marrow. Basically, the bone marrow is to be considered as OAR in RLT. However, in many patients treated with 177Lu-labeled PSMA targeting radioligands the dose of 2Gy, which is considered critical in RLT, is not reached. Studies by Scarba et al. [60] and Kabasakal et al. [61] showed that activities between 45 GBq to 65 GBq can be considered safe in a clinical context. In addition, very recently published results of a phase III study also showed that hematotoxicity occurred only in a small proportion of the treatment group [62]. However, in case of an intensively pre-treated patient or those presenting intensive involvement of the bone marrow dedicated approaches for exact dosimetry are recommended [63,64].

In addition to the aforementioned limitations due to the small sample size (especially in late cycles) another limitation of our study is the fact that we could only perform imaging until time-point 72 h p.i. for logistical reasons. However, from our point of view, the influence of the short time frame on the accuracy of our results can be neglected as the clearance of the kidneys is much faster than for DOTATATE-therapies and tumor lesions.

Some authors suggested the use of an averaged standardized half-life, e.g., Garske et al. [26]. However, as it has been shown recently by Willowson et al. [29] that the use of averaged half-life is not optimal and not an option for individualization, we decided to not use this approach. Additionally, the use and clinical evaluation of dosimetric schemes based on imaging at one single time-point within all cycles as proposed by Hänscheid et al. or Madsen et al. [27,30,33] were beyond the scope of this study.

In sum, the use of method 2 can be recommended as an appropriate alternative meeting clinical requirements especially in patients with poor health conditions, allowing for personalized dosimetry and therapy, and keeping doses to OAR within recommended limits. Assuming a dose-response relationship, personalized dosimetry may allow for applying increased activities of [^177^Lu]Lu-PSMA-617 resulting in higher tumor doses influencing tumor response rates and potentially patients’ outcome [9,11].

Additionally, considering future perspectives of PSMA-targeted radioligand therapy as a therapeutic option for metastasized hormone-sensitive prostate cancer patients (e.g., Clinical Trials.gov Identifier NCT04720157) cumulative radiation exposure and doses to organs at risk will be of even higher relevance taking into account patients’ age, life expectancy and potentially higher number of PSMA-targeted therapy cycles in this patient group. In this setting, it is therefore important to keep doses to critical organs within acceptable limits.

## 5. Conclusions

Calculation of the absorbed doses for OAR in [^177^Lu]Lu-PSMA-617 therapy using simplified dosimetric schemes is feasible and easy to implement in clinical routine. Dosimetry based on quantitative imaging at 48 h p.i. gives reliable results and allows for efficient estimation of the dose to critical organs. Furthermore, the use of only one full dosimetric calculation at cycle 1 and the extrapolation of the expected *AD* of the OAR for the following therapy cycles is possible. However, accuracy is limited and one has to keep in mind, that *AD* of the OAR might be underestimated. In general, both proposed methods allow for acceptable estimation of the expected doses to kidneys and parotid glands thus giving the opportunity to individually tailor treatment.

## Figures and Tables

**Table 1:** Characteristics of the included patient data.

Parameter		Value	
Age (years)	Mean ± SD	71.7 ± 7.9	
	Median Range (min–max)	72 53–85	
Previous systemic treatments	Chemotherapy NAAD Chemotherapy and NAAD	3 6 36	6.5% 13.0% 78.3%
	Other Additional Radium-223-dichlorid	3 3	6.5% 6.5%
Initial PSA (ng/mL)	Mean ± SD	127.1 ± 33.4	
	Median Range	74 0.5–387	
Primary Gleason Score	Mean ± SD Median Range	7.4 ± 1.4 8 3–9	
ECOG	0	20	43.5%
	1	24	52.2%
	2	2	4.3%
eGFR (mL/min/1.73 m^2^) (pretherapeutic)	Mean ± SD		82.0 ± 14.2
	Median Range (min–max)		83.0 43.4–109.4

SD: standard deviation, ECOG: Eastern Cooperative Oncology Group, NAAD: neoadjuvant androgen deprivation, eGFR: estimated glomerular filtration rate (CKD-EPI formula).

**Table 2:** Overview of gamma cameras used and the specific acquisition and reconstruction protocols.

System	Siemens Symbia T6	Picker/Philips IRIX	GE Discovery 870 CZT
	Dual head SPECT/CT	Triple-head gamma camera	Dual head SPECT/CT
Collimator	MELP	MEGP	WEHR45
Main Energy Peak	208 keV ± 7.5%	208 keV ± 10%	113 keV ± 7.5%
Whole Body Imaging	matrix 1024 × 256 scan speed: 15 cm/min	matrix 1024 × 256 scan speed: 10 cm/min	matrix 1024 × 256 scan speed: 15 cm/min
SPECT	# of proj: 120 matrix: 128 × 128 acq dur: 15 s	# of proj: 64 matrix: 64 × 64 acq dur: 15 s	# of proj: 120 matrix: 128 × 128 acq dur: 15 s
CT	120 kV, 50 mAs (DOM), 3 mm slices		120 kV, 40 mAs (DOM, ASiR reconstruction), 3 mm slices
SPECT-Reconstruction	Hermes Hybrid Recon 3.0 (3D OSEM reconstruction, AC, RR, SC)		
	5it 15ss no post filter	6it 8ss Post-filter: Butterworth (0.9 cm FWHM)	5it 15ss no post filter
SPECT Calibration Factor	9.8 cps/MBq	6.2 cps/MBq	14.3 cps/MBq

DOM: Dose Modulation; ASiR: Adaptive Statistical Iterative Reconstruction; AC: Attenuation Correction; RR: Resolution Recovery; SC: Monte-Carlo-based Scatter Correction.

**Table 3:** Overview of *eHL*, *AD*s and doses rates for kidneys and parotid glands of each cycle using full dosimetry scheme (method 1). Data are given as mean ± SD (min to max).

	Kidneys			Parotid Glands		
Cycle	Effective Half-Life [h]	Absorbed Dose [Gy]	Dose Coefficient [Gy/GBq]	Effective Half-Life [h]	Absorbed Dose [Gy]	Dose Coefficient [Gy/GBq]
1	38.2 ± 14.7 (16.1 to 72.6)	2.9 ± 1.3 (1.8 to 8.6)	0.49 ± 0.22 (0.30 to 1.44)	34.2 ± 13.3 (13.8 to 58.4)	4.8 ± 2.2 (1.2 to 9.0)	0.79 ± 0.37 (0.19 to 1.50)
2	39.1 ± 15.3 (13.2 to 83.2)	3.3 ± 1.4 (1.6 to 9.0)	0.55 ± 0.23 (0.28 to 1.51)	35.8 ± 14.0 (14.5 to 60.0)	4.6 ± 2.1 (0.8 to 9.9)	0.77 ± 0.35 (0.18 to 1.59)
3	38.7 ± 14.9 (16.9 to 75.8)	3.5 ± 1.3 (1.8 to 7.2)	0.59 ± 0.21 (0.30 to 1.21)	34.0 ± 13.8 (12.6 to 67.3)	5.3 ± 2.5 (1.2 to 11.0)	0.86 ± 0.40 (0.22 to 1.69)
4	40.0 ± 13.7 (15.2 to 62.8)	3.4 ± 0.8 (2.1 to 4.9)	0.57 ± 0.13 (0.36 to 0.83)	30.5 ± 10.9 (14.9 to 54.0)	4.8 ± 2.1 (1.3 to 8.4)	0.81 ± 0.33 (0.23 to 1.29)
5	41.5 ± 12.7 (22.4 to 61.5)	3.5 ± 0.9 (1.5 to 4.8)	0.59 ± 0.16 (0.26 to 0.81)	33.8 ± 12.9 (17.3 to 62.1)	4.3 ± 2.2 (1.2 to 8.7)	0.72 ± 0.36 (0.22 to 1.37)
6	40.9 ± 14.4 (17.5 to 63.4)	3.7 ± 0.8 (2.5 to 5.2)	0.62 ± 0.14 (0.42 to 0.86)	33.9 ± 12.2 (15.1 to 54.5)	4.8 ± 2.2 (1.2 to 9.3)	0.81 ± 0.41 (0.21 to 1.73)

**Table 4:** Results of Bland-Altman-Analysis (method 1 as reference) for cycles 2 to 6 showing mean bias and 95% limits of agreement between method 1 and method 2 for *AD* of kidneys and parotid glands using single time-point imaging at 24, 48 and 72 h p.i. Data are given as mean ± SD (min to max of 95% limits of agreement).

	Kidneys			Parotid Glands		
Cycle	24 h	48 h	72 h	24 h	48 h	72 h
2	15.4 ± 28.5 (−40.4 to 71.2)	0.63 ± 5.90 (−10.9 to 12.2)	−3.90 ± 9.31 (−22.1 to 14.3)	−7.13 ± 22.6 (−51.5 to 37.3)	1.08 ± 7.70 (−14.0 to 16.2)	3.67 ± 14.84 (−25.4 to 32.8)
3	15.1 ± 30.4 (−44.6 to 74.7)	2.42 ± 6.61 (−10.5 to 15.4)	−2.11 ± 10.8 (−23.3 to 19.1)	13.0 ± 26.2 (−38.4 to 64.4)	−1.69 ± 7.57 (−16.5 to 13.2)	4.16 ± 8.58 (−12.7 to 21.0)
4	−11.5 ± 27.2 (−64.8 to 41.8)	2.96 ± 9.13 −14.9 to 20.9)	6.26 ± 10.1 (−13.5 to 26.0)	−6.87 ± 30.4 (−66.4 to 52.6)	−2.57 ± 6.72 (−15.7 to 10.6)	4.92 ± 11.4 (−17.5 to 27.3)
5	11.1 ± 28.0 (−43.8 to 65.9)	2.12 ± 12.0 (−21.3 to 25.6)	2.84 ± 14.3 (−25.3 to 31.0)	19.1 ± 34.3 (−48.2 to 86.4)	−2.79 ± 8.05 (−18.6 to 13.0)	−7.40 ± 12.7 (−32.4 to 17.6)
6	−6.78 ± 33.6 (−72.7 to 59.1)	−0.38 ± 15.9 (−31.5 to 30.8)	4.91 ± 21.8 (−37.8 to 47.6)	12.7 ± 27.6 (−41.4 to 66.7)	3.22 ± 13.4 (−23.1 to 29.5)	−8.11 ± 14.8 (−37.1 to 20.9)

**Table 5:** Comparison of the derived AD to kidneys and parotid glands using method 3 and mean difference to the results using method 1. Data are given as mean ± SD.

	Kidneys		Parotid Glands	
Cycle	Mean *AD* [Gy]	Mean Difference to M1 [%]	Mean *AD* [Gy]	Mean Difference to M1 [%]
1	2.98 ± 1.32	0.00 ± 0.00	4.77 ± 2.21	0.00 ± 0.00
2	2.91 ± 1.25	−10.30 ± 18.50	4.71 ± 2.20	2.30 ± 11.79
3	2.90 ± 1.09	−16.03 ± 16.90	5.00 ± 2.31	−3.81 ± 16.31
4	2.94 ± 0.78	−12.43 ± 21.28	4.67 ± 2.06	−2.49 ± 17.18
5	2.81 ± 0.48	−15.29 ± 22.90	4.50 ± 1.95	8.80 ± 18.35
6	3.00 ± 0.51	−17.89 ± 16.10	4.73 ± 2.00	0.13 ± 13.73

## Data Availability

The data presented in this study are available on request from the corresponding author. The data are not publicly available due to privacy regulations.
